# Comparison of Accessibility among Vision-impaired Patients Visiting Mobile and Stationary Hospitals in Rural Bangladesh

**DOI:** 10.3329/jhpn.v31i2.16387

**Published:** 2013-06

**Authors:** Md. Ferdaws Alam, Virasakdi Chongsuvivatwong, Hasib Mahmud, Pradip Sen Gupta

**Affiliations:** ^1^Impact Foundation Bangladesh, 3rd Floor, Cosmopoliton Centre, House 22/2, Block B, Babor Road, Muhammadpur, Dhaka 1207, Bangladesh;; ^2^Epidemiology Unit, Faculty of Medicine, Prince of Songkla University, Hat Yai, Songkla 90110, Thailand

**Keywords:** Health science accessibility, Mobile hospital, Vision impairment, Bangladesh

## Abstract

The aim of this study is to compare accessibility of vision-impaired (VI) patients to other eyecare centres before attending the mobile and stationary hospitals. Under a cross-sectional study design, VI patients were consecutively enrolled if they visited one of the three Impact Foundation Hospitals—one mobile and two stationary hospitals. The cost and service output of all hospitals were also reviewed; 27.7% of patients at the mobile and 36.8% at the two stationary hospitals had sought eyecare at other health facilities in the past. Mobile hospital patients lived closer to the hospital but spent more time in travelling, bore less direct cost, needed less extra support, and had a higher level of satisfaction on the service. They also identified more barriers to access eyecare in the past. The mobile hospital had a higher percentage of patients with accessibility problems and should continue to help the remote population in overcoming these problems.

## INTRODUCTION

Worldwide, 285 million people are vision-impaired; about 90% of them live in developing countries (1)—around 62 million in the South Asia region (2) and 6.65 million in Bangladesh (3). About 80% of all vision impairment (VI) are avoidable (1). People with VI are a great burden on Bangladesh, with an annual incidence of 130,000 new cases (3).

In 2009, the population of Bangladesh was approximately 146 million (4). The majority (74%) lives in rural areas, and 36% of the total population lives below the poverty-line (5). The Government provides rural health services through community clinics, union subcentres and subdistrict health complexes free of charge (6). Only 141 hospitals provide eyecare services; of them, 71, 56, and 14 are run by the Government, non-governmental organizations (NGOs), and private sectors respectively. Eyecare services are virtually non-existent at the rural community and subdistrict level (7).

Impact Foundation Bangladesh (IFB) is a charitable, non-governmental organization which was initiated in 1993. Its mission is to prevent disability by improving the living conditions of disadvantaged people and communities. Impact ‘Jibon Tari’ (IJT) Floating Hospital was the first and unique project of its kind in Bangladesh, starting its journey in April 1999. The hospital floats along the riverside of the country and moves to a new site every 5-6 months. It provides specialized curative health services, with special emphasis on avoidable disabilities in rural areas. About 60% of outpatients have eye problems, and more than 80% of all operations performed there involve the eyes.

The Foundation also initiated Impact Masudul Haque Memorial Community Health Centre (IMCHC) in April 2002 in Chuadanga district. It provides multiple services, including eyecare to the poorer communities. Impact ‘Jibon Mela’ (IJM) is another comprehensive programme of IFB, located in Meherpur district, with similar activities. It was inaugurated in October 2007. Since their inception, IMCHC and IJM have conducted 7,481 and 1,846 eye surgeries respectively.

Two-thirds of people living in the Subcontinent do not utilize health services (8-10) due to geographical isolation (11,12). Mobile hospitals may be able to improve the geographical accessibility to health services. Previous studies in developed countries found that mobile hospitals were an effective method in reaching high-risk individuals who were not in contact with medical services (13-17). In Bangladesh, the IJT mobile hospital has been operational for 12 years and one of the two stationary hospitals under IFB for 9 years and the other one for 4 years. As the socioeconomic development of the country has been improving (18-20), there is an opportunity to see whether the mobile service is still viable.

In 2011, we conducted this study with an objective to compare previous accessibility to eyecare among vision-impaired patients of mobile and stationary hospitals, with cost aspects taken into consideration. The main rationale of mobile services worldwide is to reduce or eliminate inequity in care. In addition to having implications for future planning of IFB, the study may also serve as a model for evaluation of mobile services in developing countries where the pattern of access to eyecare is changing.

## MATERIALS AND METHODS

A cross-sectional study was conducted between May and September 2011. Data on cost and health service statistics of IFB hospitals were obtained from the IFB office, and descriptive statistics were presented.

We assumed that 90% of the mobile and 80% of the stationary hospital patients with VI had difficulty in accessing eyecare in the past. To detect this difference of 10% with an accuracy of 5% and a power of 80%, 219 subjects were required from each type of hospital. Since two stationary hospitals were included in the study, 110 subjects were recruited from each of those groups.

New patients with chief complaints of difficulty in seeing from one or both eyes and presenting with visual acuity (VA) of less than 6/24 in the worse eye (by Snellen's chart) were eligible for the study. We chose the worse eye as criterion of selection because this level of poor visual acuity would need serious medical attention, although he/she could function with the other better eye. We also used pinhole to correct for refractive error. Patients with impaired cognitive function, hearing problems, and any conditions that would disable or distort their ability to answer questions properly were excluded from the study.

A structured questionnaire was designed to collect data on previous accessibility to other eyecare services, accessibility to IFB hospitals, barriers to access other eyecare services, reasons for accessing IFB hospital services, distance from home to hospital, travel time, appointment time, travelling cost, treatment cost, extra support, and overall satisfaction with the service quality of previously-visited hospitals and IFB hospitals. The subjects were asked to choose the most important barriers to eyecare and reasons for accessing IFB hospitals. The main outcome of this study is accessibility to eyecare.

### Ethics

This study was approved by the Ethics Committee for Research in Human Subjects, Prince of Songkla University, Thailand (EC 54-202-18-5-3). Consent was taken from the authority of IFB and three project areas of IFB prior to conducting the study. Oral and written informed consent was obtained from all participants prior to data collection and eye examination, and the current study adhered to the tenets of the Helsinki Declaration.

### Statistical analysis

Data were entered and validated with Epidata program (version 3.1) (The EpiData Association, Odense, Denmark). R software (version 2.13.1) (R Foundation, Vienna, Australia), and Epicalc package was used for statistical analysis. Median (IQR) and frequencies were used for descriptive statistics. For inferential statistics, the Ranksum test was used for comparison of continuous variables not normally distributed while the chi-square test was used for comparison of categorical variables. The level of statistical significance was set at p<0.05.

## RESULTS

Table 1 displays statistics of services and expenditure of various activities of IFB hospitals. While total patients were not so much different between IJT (mobile) and IMCHC (one of the stationary hospitals), treatment and surgical cost per patient at the former was half of that at the latter. The directions of the difference for other items were similar. Thus, from the IFB perspective, the efficiency of this mobile hospital was comparable to that of a stationary hospital.

**Table 1a.** Statistics of Impact Foundation Bangladesh for 2010 and 2011

Place of service	Type of service	2010			2011		
		IJT (n)	IMCHC (n)	IJM (n)	IJT (n)	IMCHC (n)	IJM (n)
Outpatients	Total	38,867	39,999	26,453	34,978	31,521	24,041
	Eye service	22,928	-	-	20,379	-	-
In-patients	Total	2,161	1,825	1,026	2, 427	1,300	886
	Eye surgery	1,778	1,383	766	2, 047	1,039	670
Outreach activities	Training provided to birth attendants	90	348	71	73	300	77
	Attendees to mother club meeting	-	10,655	3,462	-	8,942	2,661
	Health education	41,667	29,160	52,726	37,102	24,837	45,762
	Home-gardening	-	1,462	206	-	1,067	181

**Table 1b.** Statement of expenditure of hospitals in 2011

Type of expenditure	IJT BDT (%)	IMCHC BDT (%)	IJM BDT (%)
Treatment and surgery	8,926,012 (50)	13,337,306 (45)	6,919,618 (49)
Hospital maintenance	1,199,860 (6.8)	1,481,923 (5)	861,422 (6.1)
Field programme	3,748,925 (21)	6,816,845 (23)	2,965,551 (21)
Staff salaries	3,052,696 (17)	5,365,827 (18)	2,541,900 (18)
Administration	357,040 (2)	1,012,586 (3.5)	282,433 (2)
Vehicle fuel	267,781 (1.5)	949,928 (3.2)	303,616 (2.2)
Vehicle maintenance	128,942 (0.7)	377,658 (1.3)	174,532 (1.2)
Staff development	170,768 (1)	296,385 (1)	72,597 (0.5)
Total expenditure	17,852,024 (100)	29,638,458 (100)	14,121,669 (100)
Treatment and surgical cost per patient	238	406	277

IJT=Impact ‘Jibon Tari’ Floating Hospital; IMCHC=Impact Masudul Haque Memorial Community Health Centre; IJM=Impact ‘Jibon Mela’

Table 2 shows a comparison of demographic characteristics by type of hospital. There were more males than females, and almost all were Muslim. The majority of patients were aged over 60 years and married. The median monthly family income was BDT 5,000-8,000. Thus, more than half of patients in all three hospitals lived below the poverty-line, defined by World Bank as income less than US$ 1.25 per person per day. Overall, the stationary hospitals were more likely to serve the poorer socioeconomic group.

**Table 2.** Comparison of demographic characteristics of participants between the mobile and the two stationary hospitals of Impact Foundation Bangladesh

Demographic characteristics	Type of hospital		p value
	Mobile	Stationary	
Age in years^*^	60 (49.5, 65)	60 (48, 65)	0.637
Sex			0.207
Male	138 (62.7)	124 (56.4)	
Female	82 (37.3)	96 (43.6)	
Religion			1
Muslim	212 (96.4)	213 (96.8)	
Others	8 (3.6)	7 (3.2)	
Marital status			0.033
Single	1 (0.5)	9 (4.1)	
Married	183 (83.2)	180 (81.8)	
Widowed	36 (16.4)	31 (14.1)	
Education level			0.311
No formal education	127 (57.7)	141 (64.1)	
Primary school	39 (17.7)	30 (13.6)	
Secondary/Vocational school	43 (19.5)	34 (15.5)	
College and higher	11 (5)	15 (6.8)	
Occupation			<0.001
Unemployed	50 (22.7)	96 (43.6)	
Farming	83 (37.7)	36 (16.4)	
Others	41 (18.6)	40 (18.2)	
Housewifery	46 (20.9)	48 (21.8)	
Family members^*^	5 (4, 7)	4 (2, 5.2)	<0.001
Family income^*^ (BDT)	8000 (4000, 15000)	5000 (3000, 10000)	<0.001

*Continuous variables; Median (IQR) were used for continuous variables; IQR=Interquartile range; Others are categorical variables; Number (percentage) were used for categorical variables

Table 3 shows a comparison of the effect of VI on patient's life, clinical history of chronic disease, and severity of VI. Median time since the first feeling of vision-impairment, the first feeling that vision-impairment hampered daily activities, and the first thoughts of consulting a doctor were not significantly different between types of hospital. Most parameters on VI were similar, though visual acuity of the mobile hospital patients who had never sought eyecare in the past was slightly worse. Patients at the stationary hospitals were more likely to be diabetic and have a history of eye surgery.

**Table 3.** Comparison of the effects of vision impairment on patient's life, severity of vision impairment, and clinical history of chronic disease

Effect of vision impairment on patient's life	Type of hospital		p value
	Mobile	Stationary	
Months since first felt vision-impaired^*^	36 (24,60)	36 (18,72)	0.966^†^
Months since VI hampered daily work^*^	12 (12,25.5)	12 (6,24)	0.01^†^
Months since first wanted to consult doctor^*^	12 (6,24)	12 (3.8,24)	0.298^†^
Severity of visual impairment			
Median visual acuity among all patients^*^	6/36 (6/33, 6/60)	6/36 (6/24, 6/60)	0.216^†^
Visual acuity among patients who had visited any eyecare for the first time^*^	6/36 (6/36, 6/60)	6/36 (6/24, 6/60)	0.034^†^
Clinical history of chronic disease			
Hypertension	18 (8.2)	25 (11.4)	0.335
Diabetes mellitus	6 (2.7)	18 (8.2)	0.021
Eye surgery	16 (7.3)	31 (14.1)	0.031

^*^Continuous variables; Median (IQR) were used for continuous variables; IQR=Interquartile range; Others are categorical variables; Number (percentage) were used for categorical variables;

^†^Ranksum test, others are chi-square test

Table 4 displays accessibility to other eyecare centres in the past and IFB hospitals in the current visit; 27.7% of patients at mobile and 36.8% at stationary hospitals had visited other eyecare centres in the past. There was little evidence that either group had more problem of access to eyecare in the past, although patients visiting mobile hospital lived, on average, at further distance from the eyecare centre. A significant difference was observed in relation to accessibility to the current hospital. Patients at the mobile hospital lived closer to the hospital but spent more time in travelling, bore less direct cost, needed less extra support, and had a higher level of satisfaction from the service.

**Table 4.** Accessibility to other eyecare centres in the past and IFB hospitals in the current visit

Accessibility to other eyecare centres	Type of hospital		p value
	Mobile (n=61)	Stationary (n=81)	
Ever visited eyecare centre	61 (27.7)	81 (36.8)	0.053
Distance to the last-visited eyecare centre^*^ (km)	22 (10,52)	17 (5,40)	0.211^†^
Travel time^*^ (hr)	1.5 (0.8,3)	1 (0.5,2)	0.019^†^
Waiting time^*^ (hr)	0.8 (0.2,1.5)	0.7 (0.3,1.2)	0.909^†^
Indirect cost^*^ (BDT)	50 (20,120)	40 (10,100)	0.083^†^
Direct cost^*^ (BDT)	70 (30,300)	200 (50,300)	0.089^†^
Extra support	33 (54.1)	54 (66.7)	0.178
Satisfaction with service^‡^	35 (57.4)	38 (46.9)	0.287

Accessibility to IFB hospitals	Mobile (n=220)	Stationary (n=220)	
Distance to the nearest eyecare centre^*^ (km)	17.5 (11,25)	13.5 (5,19)	<0.001^†^
Distance to IFB hospital^*^ (km)	11 (5,18.5)	14 (6,20)	0.047^†^
Travelling time^*^ (hr)	1 (0.7,2)	1 (0.5,1.2)	<0.001^†^
Waiting time^*^ (hr)	1.6 (0.8,2.5)	1.2 (0.6,2.5)	0.073^†^
Indirect cost^*^ (BDT)	25 (10,50)	30 (10,51.2)	0.349^†^
Direct cost^*^ (BDT)	20 (20,20)	20 (20,20)	<0.001^†^
Need extra support	123 (55.9)	156 (70.9)	0.002
Satisfied with service^††^	191 (86.8)	165 (75)	0.002

^*^Continuous variables; Median (IQR) were used for continuous variables; IQR=Interquartile range; Others are categorical variables; Number (percentage) were used for categorical variables;

^†^Ranksum test; others are chi-square test;

^‡^Satisfied with service” refers to “I was satisfied with the service I received, after I had my treatment”;

^††^“Satisfied with service” refers to “Before I had my service, I came to the IFB hospital because I thought I would be satisfied with the treatment”

Table 5 shows the barriers and reasons for accessing eyecare services. The percentage of patients identifying a barrier to eyecare access in the past was less in the stationary hospitals. Overall, cost was the main barrier to accessing other eyecare centres, which motivated the patients to seek cheaper services at both types of IFB hospitals. Distance was not the main barrier to accessing other eyecare centres, although more than 52.7% said it was the main reason for coming to the mobile hospital.

**Table 5.** Barriers to and reasons for accessing eyecare services

Barrier to other eyecare services	Type of hospital	
	Mobile N (%)	Stationary N (%)
Unable to pay direct costs	124 (56.4)	96 (43.6)
Unable to pay indirect costs	102 (46.4)	20 (9.1)
Quality of service not good enough	47 (21.4)	96 (43.6)
Distance too long	28 (12.7)	11 (5)
Unable to come alone	24 (10.9)	5 (2.3)
Travelling time too long	20 (9.1)	5 (2.3)
Waiting time for appointment too long	12 (5.5)	2 (0.9)
Waiting time for consultation too long	9 (4.1)	4 (1.8)
Reasons for accessing IFB hospital service		
Able to pay direct cost	148 (67.3)	103 (46.8)
Satisfied with quality of service§	147 (66.8)	181 (82.3)
Able to pay indirect cost	121 (55.0)	13 (5.9)
IFB hospital is nearer	116 (52.7)	12 (5.5)
Travelling time is short	91 (41.4)	4 (1.8)
Able to come alone	13 (5.9)	0 (0)
Short waiting time for appointment	9 (4.1)	1 (0.5)
Short waiting time for consultation	8 (3.6)	1 (0.5)

^§^“Satisfied with service” refers to “Before I had my service, I came to the IFB hospital because I thought I would be satisfied with the treatment”

## DISCUSSION

The results suggest that the mobile and stationary hospitals had comparable efficiency in terms of the number of performed surgeries vs costs. All three hospitals serve mainly the poor rural residents predominated by males whose activities were hampered by VI and had been waiting for about one year for consultation. Around one-third ever sought eyecare services elsewhere. Their main barrier to accessing eyecare was more financial than geographical.

Our data showed that the proportion of the patients who had previously accessed eyecare was not statistically significant. This may suggest that mobile service does not always serve the rural population. From the social perspective, mobile services could be efficient if they could visit pockets of untreated cases (21) in areas where healthcare facilities are unavailable. Currently, the mobile hospital has more difficulties in accessing the interior areas of the country, which is gradually becoming inaccessible due to shrinkage of river-ways and loss of waterbodies (22,23). Land transportation is becoming easier due to improvement of roads and highways. For patients in areas accessible by road, the emphasis should be on quality of care (24) rather than geographical accessibility.

The gender issue plays a key role in access to healthcare in developing countries, like Bangladesh (25,26). The Bangladesh national blindness and low vision survey in 2000 revealed that the prevalence of cataract, the main cause of vision impairment, is slightly higher among females (3). Our study shows that the service recipients are mostly males, which indicates that there is still a huge unmet need for treatment of vision impairment in the rural female population (27). The main reasons for gender disparity appear to be the existing patriarchal, patrilineal, and patrilocal social customs in Bangladesh.

**Figure.**
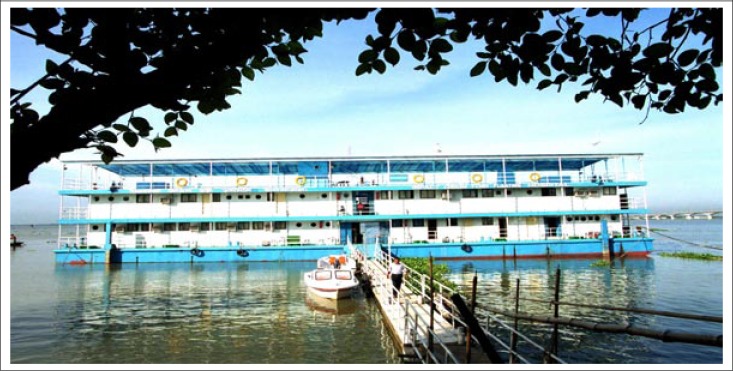
Impact ‘Jibon Tari’ Floating Hospital

Despite the difficulties in access to public facilities, such as electricity and water supply, the total running cost of IJT (mobile hospital) is still well-contained. Voluntarism spirit among permanent and visiting staff of mobile hospital allows costs to be lower than the market price. Effective public relation and patient recruitment as well as low level of charge for specialized services enable the mobile hospital to provide a relatively large volume of service per unit time compared to a multi-faceted stationary hospital (28).

The stationary service, on the other hand, is more effective and locally sustainable, especially for health promotion and prevention and people's empowerment. A high activity of such care in stationary hospitals demands higher running costs on human resources and materials and may provide less immediately-perceivable health improvement compared to curative surgery. These, however, may be more effective and efficient from the long-term societal perspective since the majority of diseases and disabilities in the rural area of the community are preventable.

The transition of mobile surgical curative care to stationary integrated care needs to be considered carefully. Our data show that geographic barriers are now a less important problem than financial barriers and perceived quality for eyecare (29-31). If data could be generalized, it would be more important currently to improve the healthcare financial system and quality of care than to aim at the geographic coverage for curative surgery. The mobile hospital, having a higher percentage of patients with accessibility problems, should continue to help remote populations in overcoming these problems.

This study has certain limitations for the fact that IFB hospitals have been there for years. Patients might select to use them due to other reasons as specified in this study. The two types being at different geographic locations would have served different populations; so, comparison must be interpreted with caution.

## ACKNOWLEDGEMENTS

This study is part of the research by the first author to fulfil the requirements for the MSc. degree in Epidemiology from the Prince of Songkla University, Thailand. The authors thank Dr. Joydhan Tanchangya, Senior Medical Officer, Impact ‘Jibon Tari’ Floating Hospital and Dr. Shaheen, Consultant, Impact Foundation Bangladesh, for advice on proposal development and diagnosis of disease. Epidemiology unit of the Prince of Songkla University is supported by the National Science and Technology Development Agency of Thailand through Research Chair Grant given to Professor Virasakdi Chongsuvivatwong (second author of this article).
